# Frequency of Hepatorenal Syndrome Among Patients With Cirrhosis and Outcome After Treatment

**DOI:** 10.7759/cureus.10016

**Published:** 2020-08-25

**Authors:** Samina Fida, Syed Murtaza S Khurshid, Hala Mansoor

**Affiliations:** 1 Medicine, CMH Lahore Medical College and Institute of Dentistry, Lahore, PAK; 2 Nephrology, Doctors Hospital and Medical Centre, Lahore, PAK; 3 Gastroenterology and Hepatology, CMH Lahore Medical College and Institute of Dentistry, Lahore, PAK

**Keywords:** hrs, hepatorenal syndrome, cirrhosis, terlipressin, sbp, spontaneous bacterial peritonitis, rfts, renal function tests, octreotide

## Abstract

Introduction

Hepatorenal syndrome is the third most common cause of admissions among patients with liver cirrhosis and has a high mortality rate. It is a progressive deterioration of renal function in a patient with acute or chronic liver failure. The only definite curative treatment of choice for hepatorenal syndrome is liver transplantation. This study aimed to determine the frequency of hepatorenal syndrome among patients with liver cirrhosis and to determine its outcome after treatment.

Patients and Methods

This case series prospective study was conducted at the Department of Medicine, CMH Lahore Medical College and Institute of Dentistry, Pakistan, from January 2019 to December 2019. The study included 136 patients of cirrhosis who were identified and worked up for hepatorenal syndrome. The patients with liver cirrhosis diagnosed as having hepatorenal syndrome were given treatment comprising injection terlipressin 2 mg four times a day and injection Haemaccel twice a day for two weeks, and after that the outcome was measured with a follow-up of six weeks.

Results

A total of 136 patients of cirrhosis were included in the study. Of the patients, 14 (10.3%) were diagnosed as suffering from hepatorenal syndrome. These diagnosed cases were given treatment for two weeks. Three (21.4%) of the patients having hepatorenal syndrome did not show any response, two (14.3%) patients recovered partially, four (28.6%) patients recovered fully, and four (28.6%) expired within one month of the treatment. One (7.14%) patient was referred during the treatment for liver transplant.

Conclusions

Hepatorenal syndrome is a common complication of cirrhosis. The treatment of systemic vasoconstrictors for hepatorenal syndrome proved to be effective in our study and should be the first priority for treating hepatorenal syndrome especially in places like Pakistan where liver transplantation is not that easily available.

## Introduction

Liver cirrhosis is the end result of persistent and chronic hepatocellular injury and can ultimately lead to hepatic dysfunction and failure. It is an irreversible pathological process featured by fibrosis and nodular regeneration. The number of deaths by this lethal disease is among the highest worldwide [1]. In Pakistan, the figure of patients with cirrhosis is quite high, and unfortunately more than 65% of the cases are due to hepatitis B and C, both of which are preventable by standard community health services [2].

The complications of cirrhosis, such as hepatic encephalopathy, upper gastrointestinal bleed, HRS and hepatopulmonary syndrome, cause a high mortality rate of the disease. Hepatorenal syndrome (HRS) is a complication describing functionally deteriorating kidneys in liver failure (either acute or chronic) [3].

The reduction in renal blood flow and glomerular filtration rate (GFR) by administration of non-hormonal anti-inflammatory drugs to cirrhotic patients with ascites was shown by Anand et al. [4]. Further studies in the following two decades demonstrated that renal failure occurred because of vasoconstriction of the renal circulation and intense systemic arteriolar vasodilatation, resulting in reduced systemic vascular resistance and arterial hypotension [5].

The HRS is a syndrome of functional renal failure due to end-stage liver disease. It is caused by impaired renal perfusion pressure, stimulation of the renal sympathetic nervous system, and production of mediators causing mesangial contraction and reduced filtration fraction. The hallmark of HRS is renal vasoconstriction and splanchnic and systemic vasodilatation [6]. Researches could refer to the main theories explaining the pathophysiological background of HRS: “Arteriolar Vasodilatation Theory” and “Hepatorenal Reflex Theory” [7].

Three important and easily recognized risk factors are low mean arterial blood pressure (<80 mmHg), dilutional hyponatremia, and severe urinary sodium retention (urine sodium <5 mEq/L). Interestingly, patients with advanced liver disease defined by a high Child-Pugh score or worsening parameters of liver function such as albumin, bilirubin, and prothrombin levels are not at a higher risk of developing HRS [8].

HRS is common, with a reported incidence of 10% among hospitalized patients with cirrhosis and ascites. The diagnosis of HRS is one of exclusion, and the International Ascites Club has devised the criteria for the diagnosis of HRS according to which four major criteria should always be fulfilled. The minor criteria have been laid down to have additional supportive evidence [9].

 Major criteria for the diagnosis of HRS devised by International Ascites Clubs are as follows:

1. Low glomerular filtration rate, as indicated by serum creatinine greater than 1.5 mg/dL or 24-hour creatinine clearance lower than 40 mL/min.

2. Absence of shock, ongoing bacterial infection, fluid losses, and current treatment with nephrotoxic drugs.

3. No sustained improvement in renal function (decrease in serum creatinine to 1.5 mg/dL or less or increase in creatinine clearance to 40 mL/min or more) following diuretic withdrawal and expansion of plasma volume with 1.5 L of a plasma expander.

4. Proteinuria lower than 500 mg/day and no ultrasonographic evidence of obstructive uropathy or parenchymal renal disease.

The treatment of HRS is difficult as is its diagnosis. No definitive curative medical treatment has so far been worked out [10]. Patients with HRS should be treated by supportive measures (blood pressure support and antibiotics), and hemofiltration should only be given if recovery of liver function is likely, either spontaneously or following liver transplantation [11]. Studies have been conducted regarding treatment; however, convincing data are awaited. Recently trials using terlipressin plus albumin and octereotid plus midodrine and albumin combination have shown some improvement in renal function, but this regimen could only buy time for liver transplantation, which is the only standard treatment of HRS [12].

The study was designed to figure out the frequency of HRS among patients with cirrhosis in Pakistan, which might show some geographical difference as the etiology of liver cirrhosis is different in different parts of the world, and the disease pattern might vary. As HRS is a diagnosis of exclusion; therefore, the study could also reveal other causes of renal failure. Preventive measures and intervention to save the kidney had also been highlighted in the study, which can help in making a future strategy for the treatment of HRS.

Types of hepatorenal syndrome

Based on the speed of onset of renal failure, two forms of HRS have been described.

Type 1 Hepatorenal Syndrome

Type1 HRS is an acute form of HRS in which renal failure occurs spontaneously in patients with severe liver disease and is rapidly progressive. It is characterized by a marked reduction of renal function, as defined by doubling of the initial serum creatinine to a level greater than 225 µM or a 50% reduction in the initial 24-hour creatinine clearance to <20 mL/min within two weeks. The development of type 1 HRS has a poor prognosis with 80% mortality at two weeks. Renal function may recover spontaneously following improvement in liver function. This is most frequently observed in acute liver failure or alcoholic hepatitis or following acute decomposition on a background of cirrhosis. These patients are usually jaundiced with a significant coagulopathy. Death often results from a combination of hepatic and renal failure or variceal bleeding.

Type 2 Hepatorenal Syndrome

Type 2 HRS usually occurs in patients with diuretic-resistant ascites. Renal failure has a slow course, in which it may deteriorate over months. It is associated with a poor prognosis, although the survival time is longer than that of patients with type 1 HRS.

The goal of this study is to determine the frequency of HRS among patients of cirrhosis visiting a tertiary care hospital and to determine the outcome of treatment in cirrhotic patients with HRS.

## Materials and methods

Study design

This is a hospital-based descriptive prospective case series study. This study was conducted at the Department of Medicine, CMH Lahore Medical College and Institute of Dentistry, Pakistan, from January 2019 to December 2019 after approval from Institutional Review Board of CMH. CMH is a tertiary care teaching hospital providing health services to large parts of urban Lahore and also rural areas around Lahore. It was a convenient non-probability sampling. The sample size was calculated using the EPI formula. Considering a prevalence of 10% (according to standard literature), the required sample size was 136 patients.

All the patients with cirrhosis aged above 14 years diagnosed on the basis of signs, symptoms, labs, and ultrasonography, and admitted directly from the outpatient department (OPD) or medical emergency were included in the study. Patients with hepatic encephalopathy being treated with nephrotoxic drugs, acute infection, hypovolemia, sepsis, and fulminant and sub-fulminant hepatic failure were excluded.

Data collection procedure

A total of 136 patients presenting to the emergency and medical OPD with cirrhosis were admitted. Labs (prothrombin time and serum albumin) and liver parenchymal changes on ultrasound were confirmed for the diagnosis of cirrhosis. Those with renal dysfunction and fulfilling the inclusion criteria were recruited for the study. Clinical as well as lab (serum creatinine more than 1.5 mg/dL) assessment was made. Written informed consent was taken from the patients. Spontaneous bacterial peritonitis (SBP) was considered as the confounding variable. Confounding variable was controlled by excluding patients with intra-abdominal infection. A proforma was filled for every patient included in the study mentioning the patient-related information (age, sex, and address) and investigations (labs and ultrasound) confirming cirrhosis and HRS along with diagnosis, treatment protocol, and hospitals stay. The outcomes of the patients with confirmed HRS measured in terms of full recovery, partial recovery, no effect, mortality, and referral were commented in the proforma.

HRS was defined as the development of renal failure in patients with cirrhosis in the absence of any other identifiable renal, pre-renal, and post-renal pathology. Biochemically, it was characterized by a marked reduction of renal function, as defined by increase in serum creatinine to a level greater than 1.5 mg/dL. Urinary protein less than 500 mg/24 hours was also one of the major criteria to confirm HRS.

The outcome of treatment was measured in terms of the following:

1. No effect: ­­­­­­­­­­­­­­­­­­­­­­­­­­­­­­­­­­­­It was measured by deteriorating (increasing) levels of serum creatinine.

2. Partially recovered: It was measured by a decrease in levels of serum creatinine but not less than 1.5 mg/dL.

3. Fully recovered: It was measured by decrease in levels of serum creatinine to less than 1.5mg/dL.

4. Expired: death of the patient.

Data analysis

The data were collected in a structured proforma and then entered into SPSS Version 20 (IBM Corp., Armonk, NY, USA) and analyzed. Frequency tables were generated for variables of interest (sex and Child-Pugh classification). Means and standard deviation of variables of interest (age and weight) were calculated. The chi-square test was used to check for statistical significance for any proportion of patients having HRS. P-value ≤ 0.05 was taken as statistically significant. The overall results were associated with findings in investigations.

Ethical considerations

All patients recruited for the study were informed about the nature of research, and written consent from the patient was taken and Ethical Review Committee of CMH Lahore was informed prior to the beginning of the study.

## Results

Out of 136 cirrhotic patients included in the study, 84 (62%) patients were males and the remaining 52 (38%) were females. The mean age of the patients recruited was 48.33 ± 8.4 years. Presenting complaints showed that 29 (21.3%) patients were admitted with symptoms of abdominal distention, 33 (24.3%) with anuria/oliguria, 30 (22.1%) with diarrhea/vomiting, 18 (13.2%) with jaundice, and 40 (29.4%) were follow-up cases. The labs confirming the diagnosis of cirrhosis were ultrasonography, total bilirubin, total serum albumin, and prothrombin time (clotting profile) (Table 1).

**Table 1:** Demographic details

	Mean ± Standard Deviation	Median	Mode
Age, years	48 ± 8.4	49	39
Bilirubin, mg/dL	2.029 ± 1.2	1.7	0.9
Albumin, g/dL	3.1 ± 0.5	3	3
Prothrombin time, seconds	17 ± 1.939	17	17
Creatinine, mg/dL	1.7 ± 0.7	1.7	2.0
24-hour urinary proteins, mg/day	121.2 ± 122.1	50	30

According to etiology, 33 patients were having hepatitis B and 42 patients were having hepatitis C. Eleven patients were having both hepatitis B and C. The remaining patients were having cirrhosis due to different etiology including autoimmune hepatitis a-1-antitrypsin deficiency, alcohol-induced cirrhosis, primary biliary cirrhosis, Wilson disease, hemochromatosis, drug-induced cirrhosis, and others.

Cirrhotic patients having deranged renal function as defined by serum creatinine of 1.5 mg/dL or more were 46 (33.8%) in number. Of the patients, 90 (66.1%) were having serum creatinine less than 1.5 mg/dL, which was considered being normal (Table 2).

**Table 2:** Serum creatinine of the patients

Creatinine	Frequency	Percent
Normal serum creatinine (0.6-1.4 mg/dL)	90	66.1
Raised serum creatinine (>1.4 mg/dL)	46	33.8
Total	136	100.0

Patients with deranged renal function were further worked up for the cause of renal dysfunction. The following four major criteria were applied to confirm the diagnosis of HRS: serum creatinine, ultrasound scan for renal parenchymal disease, 24-hour urinary protein, and routine urinary examination. Fourteen of the patients with serum creatinine more than 1.5 mg/dL did show normal renal parenchyma on ultrasonography without any evidence of obstructive uropathy, 24-hour urinary protein less than 500 mg/dL, and minimal changes on routine urinary examination.

Out of 46 patients, 14 (10.3%) were diagnosed as having HRS on the basis of criteria devised by the International Club of Ascites. The remaining 32 were having renal function disturbance due to other causes, which included analgesic nephropathy, hypovolemia, primary renal disease, and SBP. SBP was considered as the confounding variable and comprised 20 (14.7%) patients out of the 32 patients with renal dysfunction.

Among 136 patients with liver cirrhosis, 14 were diagnosed as having hepatorenal syndrome (Table 3). Therefore, the counted frequency of hepatorenal syndrome in the study was 10%. Regarding modified Child-Pugh classification, only 3 (2%) patients belonged to Child-Pugh class A, 89 (65%) to Child-Pugh class B, and 44 (33%) to Child-Pugh class C. Among the patient with HRS, one (7.1%) belonged to Child-Pugh class A, nine (64.3%) to Child-Pugh class B, and four (28.6%) to Child-Pugh class C (Table 4).

**Table 3:** Association of patient diagnosis with Child-Pugh classification

		Diagnosis of Patient		Total
		Cirrhosis	Hepatorenal Syndrome	
Child-Pugh classification of patients				
A: total numerical score of 5-6	Count	2	1	3
	% of Total	1.5%	0.7%	2.2%
B: total numerical score of 7-9	Count	80	9	89
	% of Total	58.8%	6.6%	65.4%
C: total numerical score of 10-15	Count	40	4	44
	% of Total	29.4%	2.9%	32.4%
Total	Count	122	14	136
	% of Total	89.7%	10.3%	100.0%

**Table 4:** Frequency of patient with HRS HRS, hepatorenal syndrome

HRS	Frequency	Percent
Cirrhotics without hepatorenal syndrome	122	89.7
Cirrhotics with hepatorenal syndrome	14	10.3
Total no. of cirrhotics	136	100.0

Fourteen patients with HRS were given treatment regimen of injection terlipressin 2 mg six hourly along with injection Haemaccel (colloid infusion) 500 mL twice a day for two weeks. Out of 14 patients, 3 (2.2%) did not show any response to the treatment, 2(1.5%) patients partially recovered, 4 (2.9%) of recovered fully, 4 (2.9%) patients expired within one month of the treatment, and 1 (0.7%) was referred to the liver transplant center before treatment completion. The outcome of the patients was described in terms of partially recovered (decrease of serum creatinine to 1.5 mg/dL but not less than 1.5 mg/dL), fully recovered (less than 1.5 mg/dL), and no effect (the serum creatinine remained same). These patients remained admitted in the hospital for three weeks. The final outcome was concluded in the sixth week with follow-up in the OPD (Table 5).

**Table 5:** Treatment outcome of patients with hepatorenal syndrome

Outcome	Frequency	Percentage
No treatment given	122	89.7
No effect	3	2.2
Partially recovered	2	1.5
Fully recovered	4	2.9
Expired	4	2.9
Referred	1	0.7
Total	136	100.0

## Discussion

Cirrhosis, an irreversible damage to the liver, is one of the leading causes of death worldwide. Most of the cirrhotic patients end up with HRS, a functional renal failure developing in a patient with cirrhosis [13]. A study by Amin et al. revealed that HRS acute kidney injury develops in 10-21% patients and is one of the common causes of admissions to the intensive care unit (ICU) among patients with liver cirrhosis [3].

Cirrhosis has been a very common disease in Pakistan unfortunately because of the high incidence of hepatitis B and C [14]. A study conducted in Karachi reported the frequency of HRS among patients with liver cirrhosis with ascites to be 15%. This study conducted in Karachi included 240 patients. Although a small study, but this was the first-ever research work conducted to highlight the incidence of HRS among patients with liver cirrhosis [15]. Our study, which included 136 patients, showed a frequency of 10%. The number of patients was less as compared to the study conducted in Karachi, but for more evidence-based data, a larger number of patients from whole of the Pakistan are needed.

In 1993, Ginès et al conducted a prospective study to document the incidence of HRS in Spain. The study showed that the probability of HRS occurrence was 18 % at one year and 39 % at five years [16].

In the USA, a retrospective study conducted in a tertiary care hospital reported that 40% of the patients with cirrhosis were mistakenly diagnosed to be having HRS. The reason given for the misdiagnosis was that the criteria for HRS were not developed before 1996. Besides this, many physicians were not aware of the recent criteria, and prevalence of HRS was reported to be 13-45.8% [17-19].

HRS is difficult to treat as no definitive curative medicine has been invented. Liver transplantation is the only effective treatment. Renal function in most of the successfully transplanted patients reverts. Liver transplantation is the ideal treatment for HRS but is limited due to the availability of donors. Patients with HRS have a higher risk of postoperative morbidity, early mortality, and longer hospitalization [20]. Gonwa and Wadei reported that at least one-third of patients require hemodialysis postoperatively, with a smaller percentage (5%) requiring long-term hemodialysis [21].

Because renal dysfunction is common in the first few days following transplantation, avoiding nephrotoxic immunosuppressants generally is recommended until recovery of renal function. However, the GFR gradually improves and reaches an average of 40-50 mL/min by the sixth postoperative week. The systemic and neurohumoral abnormalities associated with HRS also resolve in the first postoperative month.

Long-term survival rates are excellent, with the survival rate at three years approaching approximately 60%. This is only slightly lower than the 70-80% survival rate of transplant recipients without HRS and is markedly better than the survival rate of patients with HRS not receiving transplants, which is virtually 0% at three years [22].

Internationally, many studies and trials have been conducted to find out an effective curative treatment regimen for HRS. The use of vasoconstrictor agents is now being preferred in most of the studies. Research has proved that the vasoconstrictor agents affecting the splanchnic circulation are more effective that the vasodilator medication for renal circulation. Promising results have been reported after control trials with vasoconstrictor agents such as agonists of vasopressin V1 receptor, such as ornipressin and terlipressin, and somatostatin analogue, such as octreotide [23].

In our study, terlipressin was given in a dose of 2 mg four times a day for two weeks along with Haemaccel 500 mL as colloid for the same period as it was started in emergency as albumin was expensive and not easily available. The outcome of the treatment was defined in terms of partially recovered, fully recovered, no effect, and expired. Serum creatinine of 1.5 mg/dL was the cut-off value to describe the outcome parameters. Out of the 14 diagnosed patients of HRS, 13 remained admitted in the hospital for three weeks. One was referred earlier to a foreign country for liver transplantation.

Out of 14 patients, 4 (28%) had full recovery and 2 (14%) had partially recovery. Overall, 42% of the patients responded to the treatment. Four patients could not survive and died during the second and third week of hospital stay. One patient expired because he developed anterior wall STelevation myocardial infarction. Two of the 4 patients who expired were shifted to ICU but could not survive there because of septicemia. One patient went into hepatic encephalopathy after massive upper gastrointestinal bleeding and expired during a follow-up visit in emergency in the fifth week of the beginning of treatment.

A study of 196 patients by Wang et al. reported improvement in renal function in 58 (37.8%) patients using terlipressin and albumin. Comparing with our study, the results show that 4 (28%) out of 14 patients fully recovered. Considering the fact that artificial colloid Haemaccel was used instead of albumin, the difference in outcome in terms of full recovery can be justified. The expensive treatment and non-compliant patients with regard to follow-up were the limitations faced during the study [24].

Guevara and Ginès subsequently tried the combination of ornipressin and albumin in patients with HRS and found a marked improvement in renal function in four out of eight patients. But these four patients later on developed deterioration of serum creatinine upon treatment withdrawal. Renal vasoconstrictor antagonists have also been tried for HRS [25].

Peritoneal venous shunts avoid excessive increases in abdominal pressure, maintain volumic expansions, and stimulate through distension of the right atrium increased production of atrial natriuretic factor, producing a positive effect on HRS treatment. Maintained volumic expansion and simultaneous improvement in sinusoidal hypertension can be achieved with portosystemic shunts [26]. The severity of such patients’ conditions only allows the use of a transjugular intrahepatic portosystemic shunt, as it is less invasive than surgical shunts.

Renal dialysis support can only be used in cases in which there is a real possibility of reestablishing liver function or in which liver transplantation has been selected. Continuous hemofiltration is a better-tolerated way of providing renal support, as intermittent hemofiltration may cause hemodynamic instability and clinic worsening in some patients [27]. The molecular absorbent and recirculating system (MARS) is the most frequently used albumin dialysis system. The mean survival of patients is also significantly greater with MARS than with hemodialysis or hemofiltration [28]. HRS is a diagnosis of exclusion, and therefore it can be recommended that renal impairment should be actively sorted out and all treatable causes be effectively addressed and treated. Patients diagnosed as a case of HRS should be actively managed. All supportive measures should be instituted. Physicians should start giving treatment regime of vasoconstrictor agent as soon as the diagnosis of HRS is confirmed so that the grave outcome of HRS can be prevented till the final confirmation of liver transplantation plan [29].

## Conclusions

HRS is a common complication of renal failure in patients with liver cirrhosis. Systemic vasoconstrictors have proved a lot during recent research on their efficacy. In Pakistan, unfortunately, we lack centers for liver transplantation, but diagnosing and starting treatment at an early stage can reduce the mortality due to HRS among patients with liver cirrhosis.
